# Supplementary material for: Combining Ability and Gene Action Controlling Agronomic Traits for Cytoplasmic Male Sterile Line, Restorer Lines, and New Hybrids for Developing of New Drought-Tolerant Rice Hybrids
Source: Genes (Basel). 2022 May 19;13(5):906. doi: 10.3390/genes13050906 (PMC9141431; doi:10.3390/genes13050906)
Supplement: Supplementary file 1 [file genes-13-00906-s001.zip › genes-1691165-supplementary.pdf]

Table S1. Analysis of variance for the grain yield and its contributing traits for the studied genotypes under three irrigation regimes (6 days, 9 days, and 12 days).

S.O.V.	d.f	Days to heading (D)			Plant height (cm)			No. of Panicles/plant		
		**6D**	**9D**	**12D**	**6D**	**9D**	**12D**	**6 D**	**9 D**	**12 D**
Replications	2	0.01	0.01	0.02	6.8	6.2	3.7	1.7	3.6	2.3
Genotypes	37	86.3**	84.6**	97.6**	454.7**	193.7**	270.9**	38.9**	28.5**	30.9**
Parents (P)	13	173.9**	189.3**	216.0**	549.4**	179.8**	279.5**	8.6**	6.4**	12.0**
P. vs. Crosses	1	636.8**	303.0**	96.8**	1073.7**	615.7**	445.9**	276.2**	172.8**	35.8**
Crosses	23	12.9**	15.9**	30.6**	374.3**	183.2**	258.4**	45.7**	34.8**	41.5**
Lines	1	14.4**	19.7**	17.4**	2045.3**	1994.1**	1618.0**	4.0	7.8	26.2**
Tester	11	24.6**	30.3**	60.9**	512.7**	139.0**	356.1**	27.5**	17.1**	31.3**
Line x Tester	11	1.0**	1.2**	1.6**	83.9**	62.8**	37.1**	67.8**	54.9**	53.0**
Error	74	0.3	0.1	0.1**	9.8	12.2	7.8	2.1	2.6	1.9
**S.O.V.**	**d.f**	**Panicle length (cm)**			**No. of spikelets/ panicle**			**No. of filled grains /panicle**		
		**6 D**	**9 D**	**12 D**	**6 D**	**9 D**	**12 D**	**6 D**	**9 D**	**12 D**
Replications	2	0.7	0.2	0.2	42.9	30.9	58.4	43.7	85.9	113.0
Genotypes	37	13.8**	9.9**	11.8**	7376.4**	4916.3**	3783.1**	7085.3**	4691.6**	3486.5**
Parents (P)	13	15.0**	11.0**	10.3**	3262.2**	2545.9**	1843.2**	2871.1**	1773.6**	1369.8**
P. vs. Crosses	1	92.6**	85.9**	6.1**	92420.0**	52996.8**	41551.7**	79017.7**	45506.8**	33309.5**
Crosses	23	9.6**	6.0**	12.9**	6004.3**	4165.6**	3237.4**	6339.8**	4566.4**	3386.2**
Lines	1	13.5**	7.5*	0.6	44135.2**	32749.7**	20591.8**	36059.6**	23896.6**	10614.0**
Tester	11	17.2**	10.6**	25.5**	5174.9**	3288.4**	3488.8**	6627.4**	4877.6**	4471.9**
Line x Tester	11	1.7	1.2	1.5*	3367.3**	2444.4**	1408.4**	3350.3**	2498.0**	1643.4**
Error	74	0.9	1.1	0.7	191.9	109.9	44.7	191.4	78.1	37.8

** : Highly significant at 1% * : significant at 5
6 D: 6 Days, 9 D: 9 Days, and 12 D: 12 Days.

Table S1. Continue:.

S.O.V.	d.f	Panicle weight (g)			Spikelet fertility (%)			1000 grain weight (g)			Grain yield/plant (g)		
		6 D	9 D	12 D	6 D	9 D	12 D	6 D	9 D	12 D	6 D	9 D	12 D
Replications	2	1.3	0.9	0.3	2.1	18.7	1.6	0.2	1.0	0.1	6.9	14.6	18.5
Genotypes	37	7.7**	4.8**	3.5**	42.3**	73.7**	112.2**	7.3**	12.8**	15.7**	487.1**	302.6**	205.9**
Parents (P)	13	2.6**	1.1**	0.7**	12.8**	44.8**	94.5**	8.7**	12.9**	13.1**	150.0**	68.4**	53.0**
P. vs. Crosses	1	67.3**	50.0**	35.3**	15.7	0.8	0.04	60.9**	158.0**	233.3**	6040.4**	4346.2**	1978.6**
Crosses	23	8.0**	4.9**	3.7**	60.0**	93.3**	127.0**	4.1**	6.4**	7.7**	436.2**	259.2**	215.3**
Lines	1	12.3**	7.4**	8.7**	11.3	39.0	231.4**	22.1**	4.4**	7.8**	22.8	0.7	12.3
Tester	11	10.4**	5.9**	4.7**	99.1**	145.8**	165.2**	5.2**	8.1**	10.1**	381.2**	257.7**	324.5**
Line x Tester	11	5.3**	3.7**	2.3**	25.5**	45.7**	79.4**	1.5**	4.9**	5.2**	528.9**	284.1**	124.6**
Standard Error	74	0.5	0.4	0.3	4.6	8.3	5.7	0.3	0.3	0.2	22.2	18.6	12.1

** : Highly significant at 1% * : significant at 5%

6 D: 6 Days, 9 D: 9 Days, and 12 D: 12 Days.

Table S2. Analysis of variance for the grain quality traits for the studied genotypes under three irrigation regimes.

S.O.V.	d.f	Hulling (%)			Milling (%)			Head rice (%)		
		6 D	9 D	12 D	6 D	9 D	12 D	6 D	9 D	12 D
Replications	2	1.8	1.9	0.6	1.9	1.9	1.4	0.5	24.7	17.3
Genotypes	37	3.3**	3.3**	5.1**	13.4**	15.4**	17.2**	141.6**	144.1**	215.1**
Parents (P)	13	3.0**	2.6**	3.3**	5.9**	6.6**	13.3**	49.6**	51.5**	156.7**
P. vs. Crosses	1	11.6**	27.3**	39.8**	2.6	10.3**	21.7**	400.7**	211.4**	62.9**
Crosses	23	3.0**	2.7**	4.6**	18.2**	20.6**	19.3**	182.3**	193.5**	254.7**
Lines	1	1.1	0.4	0.01	209.4**	221.8**	118.5**	1247.3**	1444.7**	1411.6**
Tester	11	3.5*	2.3	6.8**	11.5**	15.5**	18.9**	149.7**	154.2**	269.9**
Line x Tester	11	2.7**	3.3**	2.8**	7.5**	7.4**	10.6**	118.1**	119.1**	134.4**
Standaed Error	74	0.9	1.0	0.8	1.1	0.7	0.7	1.8	10.3	6.8

** : Highly significant at 1% * : significant at 5%

6 D: 6 Days, 9 D: 9 Days, and 12 D: 12 Days.

Table S3. Ratio between K^2 GCA and K^2 SCA for the grain yield and its contributing traits for the studied genotypes under three irrigation regimes.

Traits	Days to heading			Plant height			No. of panicles/plant			Panicle length			No. of spikelets/ panicle		
Genetic components	**6D**	**9D**	**12D**	**6D**	**9D**	**12D**	**6D**	**9D**	**12D**	**6D**	**9D**	**12D**	**6D**	**9D**	**12D**
K^2 GCA	0.39	0.55	0.48	56.5	55.1	44.7	0.1	0.1	0.7	0.3	0.18	0.0	1220.6	906.7	570.8
K^2 SCA	4.1	5.0	10.1	83.8	21.1	58.1	4.2	2.4	4.9	2.7	1.59	4.1	830.5	529.8	574.0
K^2 GCA / K^2 SCA	0.2	0.4	0.5	24.7	16.8	9.8	21.9	17.4	17.0	0.3	0.04	0.3	1058.5	778.2	454.6
Traits	**No. of filled grains /panicle**			**Panicle weight**			**Spikelet fertility**			**1000 grain weight**			**Grain yield/plant**		
Genetic components	**6D**	**9D**	**12D**	**6D**	**9D**	**12D**	**6D**	**9D**	**12D**	**6D**	**9D**	**12D**	**6D**	**9D**	**12D**
K^2 GCA	996.3	661.6	293.8	0.3	0.2	0.2	0.2	0.9	6.3	0.6	0.1	0.2	0.02	-0.5	0.01
K^2 SCA	1072.7	799.9	739.0	1.7	0.9	0.7	15.7	22.9	26.6	0.8	1.3	1.6	59.8	39.9	52.1
K^2 GCA / K^2 SCA	1053.0	806.6	535.2	1.6	1.1	0.7	6.9	12.5	24.6	0.4	1.5	1.7	168.9	88.5	37.5

Table S4. Ratio between K^2 GCA and K^2 SCA for the grain yield and its contributing traits for the studied genotypes under three irrigation regimes.

Traits	Hulling			Milling			Head rice		
Genetic components	**6D**	**9D**	**12D**	**6D**	**9D**	**12D**	**6D**	**9D**	**12D**
σ^2 GCA	0.01	-0.02	-0.02	5.8	5.8	3.3	34.6	39.8	39.0
σ^2 SCA	0.4	0.2	1.0	1.7	1.7	3.0	24.6	24.0	43.9
K^2 GCA / K^2 SCA	0.6	0.8	0.7	2.1	2.1	3.3	38.8	36.3	42.5

Table S5. Mean performance for the grain yield and its contributing traits of the studied genotypes under three irrigation regimes.

Traits / Genotypes	1000 grain weight (g)			Grain yield /plant (g)			RYI	
	6D	9D	12D	6D	9D	12D	9D	12D
L1*	26.3	23.3	20.3	32.8	26.0	21.2	20.7	35.4
L2*	25.6	22.8	20.3	41.5	29.6	25.6	28.7	38.3
T1	26.0	22.0	18.9	55.1	41.5	26.1	24.7	52.6
T2	27.2	24.1	22.4	53.4	38.1	30.9	28.7	42.1
T3	25.3	21.3	21.8	48.4	34.1	33.1	29.5	31.6
T4	26.1	23.5	22.6	52.4	36.9	30.3	29.6	42.2
T5	25.8	24.4	22.3	53.3	37.7	31.1	29.3	41.7
T6	25.0	22.0	17.9	39.4	29.4	24.9	25.4	36.8
T7	27.0	25.4	22.1	41.7	34.3	22.0	17.7	47.2
T8	27.3	24.2	22.1	42.1	34.4	23.6	18.3	43.9
T9	27.2	25.3	22.5	43.1	32.3	26.5	25.1	38.5
T10	24.5	22.9	23.3	49.1	31.1	21.0	36.7	57.2
T11	26.9	23.7	21.8	55.8	37.4	24.9	33.0	55.4
T12	20.7	17.1	16.0	40.6	25.1	19.9	38.2	51.0
L1 × T1	26.0	23.7	21.4	50.2	38.0	23.7	24.3	52.8
L2 × T1	26.0	23.7	21.4	59.1	44.3	28.1	25.0	52.5
L1 × T2	28.6	26.2	24.9	74.5	53.9	33.4	27.7	55.2
L2 × T2	26.0	23.7	22.6	60.3	46.3	46.8	23.2	22.4
L1 × T3	28.3	27.3	25.1	73.0	56.1	40.7	23.2	44.2
L2 × T3	26.8	25.7	24.5	59.1	44.1	43.5	25.4	26.4
L1 × T4	29.1	26.8	25.5	66.2	52.1	40.7	21.3	38.5
L2 × T4	27.5	25.3	24.3	67.4	47.5	38.8	29.5	42.4
L1 × T5	27.3	25.8	23.9	75.2	55.7	43.5	25.9	42.2
L2 × T5	27.3	26.3	24.0	38.7	28.0	23.3	27.6	39.8
L1 × T6	28.7	24.9	21.0	50.4	38.3	33.1	24.0	34.3
L2 × T6	28.1	27.8	25.4	78.9	60.8	29.7	22.9	62.4
L1 × T7	28.6	26.2	25.8	75.4	53.5	34.4	29.0	54.4
L2 × T7	28.3	27.9	25.1	70.3	55.2	25.0	21.5	64.4
L1 × T8	27.4	25.4	25.5	60.1	49.4	33.5	17.8	44.3
L2 × T8	27.0	26.9	24.9	67.7	49.8	35.9	26.4	47.0
L1 × T9	28.2	26.4	26.2	59.3	51.8	51.8	12.6	12.6
L2 × T9	25.5	24.1	23.5	68.4	53.5	46.9	21.8	31.4
L1 × T10	28.5	26.7	26.1	48.3	35.0	28.0	27.5	42.0
L2 × T10	26.4	24.7	24.2	80.6	56.1	40.2	30.4	50.1
L1 × T11	27.5	24.2	22.9	43.7	31.2	23.7	28.6	45.8
L2 × T11	27.4	24.1	22.8	47.1	36.1	23.7	23.4	49.7
L1 × T12	26.0	24.7	23.6	54.0	40.7	31.4	24.6	41.9
L2 × T12 ‡	24.6	22.2	21.3	46.3	31.9	26.1	31.1	43.6
L.S.D 5%	0.8	0.8	0.7	6.6	6.1	4.9		
L.S.D 1%	1.0	1.1	0.9	8.8	8.1	6.5		

L.S.D.: Least significant difference. ‡: Control hybrid.

L1* and L2* : data recorded on the maintainer line

Table S6. Mean performance for the grain yield and its contributing traits of the studied genotypes under three irrigation regimes.

Traits / Genotypes	Hulling (%)			Milling (%)			Head rice (%)		
	6D	9D	12D	6D	9D	12D	6D	9D	12D
L1*	79.3	78.9	78.5	68.5	68.9	67.5	56.7	56.3	50.4
L2*	80.4	79.7	79.2	69.6	70.9	68.7	62.4	62.1	55.8
T1	81.1	81.1	79.7	72.1	74.0	73.5	67.4	68.0	65.5
T2	80.1	80.5	81.0	70.8	72.4	73.6	68.2	63.7	59.4
T3	79.1	80.9	81.4	69.4	72.5	72.6	63.8	59.8	61.8
T4	80.7	80.9	81.1	72.0	73.0	73.2	67.6	62.5	56.3
T5	82.6	82.2	81.3	73.6	75.4	75.0	70.8	58.3	47.8
T6	80.8	81.6	81.7	69.7	72.4	73.9	60.4	57.2	56.1
T7	79.6	81.4	81.2	70.6	73.0	73.3	60.3	63.2	63.8
T8	79.4	81.1	80.7	71.4	72.6	72.9	67.3	53.9	42.8
T9	79.5	81.0	81.2	71.5	72.5	73.6	63.3	59.9	62.8
T10	81.7	82.6	82.6	72.5	73.4	72.9	68.6	61.3	66.7
T11	79.9	80.5	81.2	71.4	72.1	73.1	67.3	68.8	66.4
T12	80.7	81.3	81.3	72.1	72.4	70.4	68.5	62.2	57.0
L1 × T1	81.5	81.2	79.8	72.9	72.2	71.9	69.7	70.1	68.6
L2 × T1	78.1	77.8	77.0	66.3	66.1	65.8	60.0	60.9	59.0
L1 × T2	80.1	80.1	79.4	71.9	72.7	71.5	69.5	69.5	67.1
L2 × T2	78.8	79.0	78.5	69.4	70.1	68.9	57.6	54.7	41.5
L1 × T3	77.3	78.8	79.8	74.0	75.8	73.9	71.2	69.2	66.6
L2 × T3	78.8	79.9	78.5	68.4	69.3	69.9	50.3	47.5	44.7
L1 × T4	79.1	79.0	78.9	72.0	72.7	73.5	61.5	58.5	67.9
L2 × T4	79.9	80.1	80.0	70.8	71.5	71.2	61.8	58.8	66.4
L1 × T5	80.9	80.5	79.6	73.8	74.9	74.4	62.6	59.1	58.7
L2 × T5	81.5	81.1	80.1	70.3	71.0	70.5	60.7	57.4	53.7
L1 × T6	78.9	78.6	78.7	72.0	72.3	50.3	63.6	62.3	60.6
L2 × T6	79.7	79.9	80.0	69.1	69.5	68.5	55.8	54.5	53.3
L1 × T7	79.8	80.3	78.8	71.4	71.8	70.9	52.3	50.1	48.7
L2 × T7	79.5	79.9	77.9	69.0	69.5	68.6	58.0	55.9	39.5
L1 × T8	79.7	80.6	79.5	71.5	72.1	72.5	64.2	60.9	54.7
L2 × T8	78.7	79.6	79.0	70.4	70.7	71.1	43.8	40.5	37.9
L1 × T9	79.8	80.7	80.6	74.0	75.0	72.2	63.3	60.9	50.8
L2 × T9	78.9	79.0	82.1	67.2	69.1	75.9	43.2	40.8	60.9
L1 × T10	79.7	80.3	80.3	77.7	79.1	76.8	67.4	58.9	57.5
L2 × T10	80.8	81.7	81.7	72.1	72.6	70.6	66.3	57.5	54.1
L1 × T11	80.9	81.2	81.9	73.3	74.1	75.1	73.0	71.8	69.5
L2 × T11	80.1	80.4	81.1	70.4	71.2	72.1	62.9	61.7	59.2
L1 × T12	80.1	79.1	78.6	72.7	71.5	69.8	67.1	63.3	60.6
L2 × T12 ‡	80.0	80.3	79.7	72.7	71.5	69.8	65.1	56.9	54.7
L.S.D 5%	1.3	1.4	1.3	1.5	1.2	8.5	1.9	4.5	3.7
L.S.D 1%	1.7	1.9	1.7	1.9	1.6	11.3	2.5	6.0	4.9

L.S.D.: Least significant difference. ‡: Control hybrid .

L1* and L2* : data recorded on the maintainer line
